# Autoantibodies in Autoimmune Liver Disease—Clinical and Diagnostic Relevance

**DOI:** 10.3389/fimmu.2018.00609

**Published:** 2018-03-27

**Authors:** Marcial Sebode, Christina Weiler-Normann, Timur Liwinski, Christoph Schramm

**Affiliations:** ^1^1st Department of Medicine, University Medical Centre Hamburg-Eppendorf, Hamburg, Germany; ^2^Martin Zeitz Centre for Rare Diseases, University Medical Centre Hamburg-Eppendorf, Hamburg, Germany

**Keywords:** autoimmune hepatitis, primary biliary cholangitis, primary sclerosing cholangitis, antinuclear antibodies, smooth muscle antibodies, antimitochondrial antibodies, soluble liver antigen/liver–pancreas antigen, serology

## Abstract

Testing for liver-related autoantibodies should be included in the workup of patients with hepatitis or cholestasis of unknown origin. Although most of these autoantibodies are not disease specific, their determination is a prerequisite to diagnose autoimmune hepatitis (AIH) and primary biliary cholangitis (PBC), and they are components of the diagnostic scoring system in these diseases. In primary sclerosing cholangitis (PSC), on the other hand, autoantibodies are frequently present but play a minor role in establishing the diagnosis. In PSC, however, data on antibodies suggest a link between disease pathogenesis and the intestinal microbiota. This review will focus on practical aspects of antibody testing in the three major autoimmune liver diseases AIH, PBC, and PSC.

## Introduction

Testing for liver-related autoantibodies should be included in the workup of patients with hepatitis or cholestasis of unknown origin. Although most of these autoantibodies are not disease specific, their determination is a prerequisite to diagnose autoimmune hepatitis (AIH) and primary biliary cholangitis (PBC), and they are components of the diagnostic scoring system in these diseases (1, 2) (Table 1). In primary sclerosing cholangitis (PSC), on the other hand, autoantibodies are frequently present but play a minor role in establishing the diagnosis, which is based on cholangiography. In PSC, however, data on antibodies suggest a link between disease pathogenesis and the intestinal microbiota (3).

**Table 1:** Autoantibodies in autoimmune liver diseases.

Antibody	Screening test	Confirmatory test	Positive in the following liver diseases	Recommended as initial screening for AILD	Prognostic value for AILD	Target antigen
ANA	SKL IFT	HEp-2 IFT (identification of a specific pattern) ELISA, WB (for selected nuclear antigens)	AIH1 PBC PSC HBV HCV DILI NAFLD	Yes	In PBC yes (only gp210)	In AIH: chromatin, histones, centromere, ds- and ss-DNA, cyclin A, ribonucleoproteins and other nuclear antigensIn PBC: centromere, lamin-B-receptor, sp100, gp210

Anti-SMA/anti-F-actin	SKL IFT	ELISA, WB IFT on fibroblasts (VSM47)	AIH1	Yes	Unclear	(Filamentous) actin, tubulin, or intermediate filaments

Anti-LKM-1	SKL IFT	ELISA, WB	AIH2 HCV	Yes	In AIH yes	CYP 2D6

Anti-SLA/LP	ELISA, WB		AIH1	Yes	Unclear	SepSecS

Anti-LC1	SKL IFT	WB Double immunodiffusion assay	AIH2 HCV	No	Unclear	FTCD

Anti-LKM-2	WB		Tielinic acid-induced DILI	No		CYP 2C9

Anti-LKM-3	WB	ELISA	AIH2 HCV HDV	No		UGTs

p-ANCA/p-ANNA	IFT on formaldehyde-fixed neutrophils	ELISA	AIH PSC HBV HCV	No	Unclear	Unclear, tubulin-beta chain?

Anti-ASGPR	ELISA, WB		AIH PBC HBV HCV	No	Unclear	ASGPR

AMA	SKL IFT	HEp-2 IFT ELISA, WB	PBC Variant syndromes Acute liver failure	Yes	No	Lipoylated domains of E3 binding protein, various epitopes of pyruvate-dehydrogenase complex, 2-oxo-acid dehydrogenase complex, branched chain 2-oxo-acid dehydrogenase complex

Anti-Kelch-like 12-antibodies, anti-hexokinase1-antibodies	ELISA, WB		PBC	Unclear	Unclear	Kelch like 12, hexokinase E1

Anti-GP2 IgA	IFT		PSC	No	In PSC yes	Glycoprotein 2

*AIH, autoimmune hepatitis; AIH1 or 2, autoimmune hepatitis type 1 or 2; AILD, autoimmune liver diseases; AMA, antimitochondrial antibodies; ANA, antinuclear antibodies; ASGPR, asialoglycoprotein receptor; CYP, cytochrome P450; DILI, drug-induced liver injury; DNA, deoxyribonucleic acid; ds, double stranded; ELISA, enzyme-linked immunosorbent assay; FTCD, formiminotransferase cyclodeaminase; GP, glycoprotein; HBV, viral hepatitis B; HCV, viral hepatitis C; HDV, viral hepatitis D; HEp-2 IFT, indirect immunofluorescence testing on HEp-2 cells; IFT, indirect immunofluorescence testing; LC1, liver cytosol type 1; LKM, liver–kidney microsome; NAFLD, non-alcoholic fatty liver disease; SKL IFT, indirect immunofluorescence testing on rodent stomach, kidney, and liver tissue; p-ANCA, antineutrophil cytoplasmatic antibodies with a perinuclear staining pattern; PBC, primary biliary cholangitis; PSC, primary sclerosing cholangitis; SEPSECS, O-phosphoseryl-tRNA:selenocysteine-tRNA synthase; SLA/LP, soluble liver antigen/liver–pancreas antigen; SMA, smooth muscle antibodies; ss, single stranded; UGT, uridine glycuronosyl transferase; WB, western blot/immunoblot*.

In Europe, screening for liver-related autoantibodies is traditionally conducted using indirect immunofluorescence testing (IFT) on rodent tissue slices (Table 2). Cutoff levels for titers included in diagnostic scores for AIH and PBC use IFT as a standard. In other parts of the world, solid-phase test systems are widely used and to date it is unclear, whether this results in altered sensitivity or specificity of the tests applied and how these findings can be translated into diagnostic scores. IFT has the advantage of not only assessing the titer of the autoantibodies but also the fluorescence pattern, which may provide additional information on the presence, e.g., of PBC-defining antinuclear antibodies (ANA). Therefore, fluorescence pattern of ANA should always be reported together with the antibody titer. It is important to note, however, that IFT is a subjective method that requires an experienced lab technician and that international standards for testing are currently lacking. Also, titers may differ between labs due to different substrates used, and, therefore, the lab-specific reference values should be noted.

**Table 2:** Recommended serologic first line diagnostics for autoimmune liver diseases.

Test assay	Antibodies detected
SKL IFT	ANA Anti-SMA Anti-LKM AMA
HEp-2 IFT	ANA patterns indicative of antibodies to mitochondria (AMA), gp210, sp100, and centromere
ELISA or WB	Anti-SLA/LP

*AMA, antimitochondrial antibodies; ANA, antinuclear antibodies; ELISA, enzyme-linked immunosorbent assay; GP, glycoprotein; HEp-2 IFT, indirect immunofluorescence testing on HEp-2 cells; LKM, liver–kidney microsome; SMA, smooth muscle antibodies; SKL IFT, indirect immunofluorescence testing on rodent stomach, kidney, and liver tissue; SLA/LP, soluble liver antigen/liver–pancreas antigen; WB, western blot/immunoblot*.

The following review will focus on practical aspects of antibody testing in the three major autoimmune liver diseases AIH, PBC, and PSC.

## Autoantibodies in AIH

### Introduction

#### Diagnosis of AIH

Autoimmune hepatitis is a chronic inflammatory liver disease with unknown etiology (1, 4, 5). The diagnosis of AIH is based on a combination of typical findings, which are not disease specific and on the exclusion of other liver diseases, such as viral hepatitis (6–8). It is assumed that AIH is a T cell-mediated disease. However, there are hints at a relevant role for a humoral immune response in AIH pathogenesis: autoantibodies can frequently be detected, plasma cells are enriched in hepatic infiltrates and immunoglobulin G (IgG)/gammaglobulins serve as a diagnostic and disease activity markers. Nevertheless, the exact pathogenetic relevance of this humoral immune response for AIH is unclear and has not been investigated in detail (Table 1). The antigens that are targeted by autoantibodies in AIH have been identified in most cases. Still, it is uncertain whether these autoantigens play a relevant role for the induction or preservation of chronic liver inflammation in AIH.

The presence of AIH-related autoantibodies supports the diagnosis of AIH. However, most autoantibodies being associated with AIH have a relatively low disease specificity (Table 1). In the setting of acute or even chronic hepatitis of an etiology other than AIH, and even in healthy persons, liver autoantibodies can frequently be found. Besides, the presence of autoantibodies is not a prerequisite for the diagnosis of AIH: around 10–15% of patients present without known autoantibodies (“seronegative” AIH) or develop them during the course of disease after an acute onset.

#### Methods of Testing

As mentioned earlier, IFT on rodent kidney, liver, and stomach tissue and on HEp-2 cells is recommended as the standard method for the detection of liver autoantibodies (9). It is important to note that IFT testing is a subjective method and titers may vary depending on the laboratory performing the test. Antibody titers above 1:40 have a higher specificity for AIH, but it is unknown whether very high titers (e.g., >1:640) are associated with an even higher specificity. IFT enables the simultaneous analysis of most of the antibodies that are relevant for autoimmune liver diseases. HEp-2 cells with their prominent nuclei help to identify specific patterns of ANA. HEp-2 cells should not be used for initial screening for the presence of ANA since low titer antibodies can frequently be found in healthy subjects using these cells (10). Therefore, screening for ANA should be performed on rodent kidney, liver, and stomach tissue. In the case of positivity, the pattern of ANA should then be analysed on HEp-2 cells (9). The fluorescence pattern of ANA on HEp-2 cells provides additional diagnostic information: a pattern of multiple “nuclear dots” or a “nuclear rim” pattern suggest the presence of anti-sp100 or anti-gp210, respectively, and thereby hint at the diagnosis of PBC or a variant syndrome of AIH with features of PBC (see below section on autoantibodies in PBC). In Europe, there is consensus that solid-phase assays such as enzyme-linked immunosorbent assays (ELISA) or immunoblots should only be used to confirm the results of IFT, but not for initial screening of ANA. However, in the USA, ELISA are frequently used for screening purposes (4). For the diagnostic workup of autoimmune liver diseases, anti-soluble liver antigen/liver–pancreas antibodies (anti-SLA/LP) should already initially be tested for by ELISA or immunoblot since these autoantibodies cannot be detected by IFT and have high specificity for AIH.

#### Subclassification of AIH According to Autoantibody Pattern

Next to their diagnostic value, certain autoantibodies have been associated with a different clinical presentation and prognosis of AIH. These associations have led to the classification of type 1 and type 2 AIH. Type 1 AIH is the predominant type of AIH in adults and children and is defined by the presence of ANA and/or antismooth muscle antibodies (anti-SMA) (7). Anti-SMA (and especially anti-actin antibodies) have been associated with inflammatory activity of AIH in adult patients (11). However, these results still need to be validated. Type 2 AIH (about 5–10% of all AIH patients) is typically defined by anti-liver–kidney microsomal type 1 antibodies (anti-LKM-1) or, in rare cases, by anti-LKM-3 and/or anti-liver cytosol type 1 antibodies (anti-LC1) (12–14). Type 2 AIH presents at a younger age, often in childhood, and is associated with a more aggressive disease course than type 1 AIH (12, 15). It is still controversial whether the presence of anti-SLA/LP defines a third subgroup of AIH (type 3 AIH) with a potentially more aggressive clinical course or whether anti-SLA/LP-positive AIH patients experience a clinical course similar to type 1 AIH patients (16–19). In 5–10% of AIH patients, anti-SLA/LP are present in combination with ANA and/or anti-SMA. In up to 10% of AIH patients, anti-SLA/LP are the sole antibodies detected (20), a fact that mandates their testing for the diagnosis of AIH.

#### Special Considerations in Children

Antibody testing in pediatric patients demands special attention. Titers for most liver autoantibodies seem to correlate with age: in children, lower titers (1:20 for ANA and anti-SMA and 1:10 for anti-LKM-1) can be diagnostic for AIH, whereas in adults, higher titers of ANA (1:80–1:160) can also be found in healthy individuals (9, 21). Therefore, the physician should be informed by the laboratory on any antibody titer that is detected and interpret the results according to patient’s age.

#### Repeated Testing of Autoantibodies

Particularly in pediatric patients with autoimmune liver diseases, titers of liver-related autoantibodies can correlate with disease activity and response to treatment may be paralleled by reduction or even disappearance of antibody titers (21, 22). Thus, retesting of autoantibodies can be reasonable in children, as it can add information on the depth of remission achieved.

Retesting of autoantibodies for adult patients with AIH cannot be recommended in general, but can be helpful in special clinical settings. In cases of acute hepatitis and initially negative autoimmune serology, antibody testing should be repeated after 3–6 months since antibodies can appear during the course of disease. In adult patients, a variant syndrome of AIH with features of additional PBC can develop even several years after the diagnosis of classical AIH. Therefore, IFT should be repeated to screen for PBC-specific ANA or antimitochondrial antibodies (AMA) whenever cholestatic liver enzymes remain or become elevated or when symptoms suggestive of PBC develop in an AIH patient. Currently, regular retesting of autoantibodies in adult patients as a surrogate marker for inflammatory activity is not recommended.

### Antinuclear Antibodies

Antinuclear antibodies were the first autoantibodies to be associated with AIH (23). However, they lack disease specificity. About 50–75% of AIH patients are ANA-positive (with or without anti-SMA) (24). ANA can also be detected in healthy persons or patients with other liver diseases such as fatty liver disease, drug-induced liver injury (DILI) disease, or viral hepatitis. The pattern of ANA in AIH often is speckled or homogenous. It is uncertain whether a distinct pattern of ANA has a higher specificity for AIH. ANA in AIH are directed against several antigens such as chromatin, histones, centromere, double-(ds) and single-(ss) stranded deoxyribonucleic acid (DNA), cyclin A, ribonucleoproteins or other nuclear antigens that have not yet been identified (25–29). A biochemical differentiation of these antigens is not recommended because they have not been associated with a certain clinical course or a higher diagnostic specificity for AIH. It is important to acknowledge that up to 20% of AIH patients may display anti-dsDNA antibodies. This may cause confusion with the diagnosis of systemic lupus erythematodes, which has to be ruled out in patients with respective clinical findings. In rare cases, both diseases may occur together (30).

### Antismooth Muscle Antibodies

Similar to ANA, anti-SMA have been associated with AIH since the early days of the clinical definition of AIH. They are also not disease specific and can be detected in various liver diseases such as fatty liver disease (31, 32). Anti-SMA are present in about 50% of patients with type I AIH and can be the only detectable autoantibody. In IFT, anti-SMA react with the smooth muscles of the lamina propria and muscularis mucosae of the stomach or arterial walls of the liver. On kidney tissue, anti-SMA show different staining patterns: the vascular/glomerular and the vascular/glomerular/tubular (VGT) patterns are more specific for AIH than the vascular (V) pattern (9, 24). The VGT pattern can be confirmed by IFT performed on fibroblasts or vascular smooth muscle cells (VSM47) (33). On a molecular level, anti-SMA are a heterogeneous group of antibodies showing reactivity against actin, tubulin or intermediate filaments (34). Sera showing the VGT pattern react in 80% with filamentous actin (F-actin) (9, 33, 35). Actin is a ubiquitous contractile protein. The presence of anti-F-actin antibodies can be confirmed by solid-phase assays, such as ELISA (36). Anti-SMA showing reactivity against F-actin seem to be more specific for AIH, but can also be detected in other liver diseases (35, 37).

### Anti-Liver–Kidney Microsomal Antibodies (Anti-LKM)

Anti-LKM-1, but also anti-LKM-3 are defining type 2 AIH (9). In IFT, anti-LKM stain, the cytoplasm of hepatocytes and the proximal, larger renal tubuli, but not parietal cells of the stomach (38). By contrast, AMA in PBC stain the mitochondria-rich, distal, smaller renal tubuli, and gastric parietal cells. ELISA tests are recommended to confirm positivity for anti-LKM in IFT (39). The autoantigen that is recognized by anti-LKM-1 in AIH is cytochrome P450 (CYP) 2D6 (40–44). Anti-LKM-1 are not AIH-specific and can also be found in patients with chronic viral hepatitis C (HCV) (45, 46). However, the epitopes that are targeted by anti-LKM-1 in HCV-infected patients differ from those in patients with AIH (47, 48). Anti-LKM-2 have been detected in cases of tielinic acid-induced DILI, a drug that has been withdrawn from the market (49). In contrast to anti-LKM-1, anti-LKM-2 target a different isoform of the cytochrome P 450, namely CYP 2C9. Anti-LKM-3 can be found in few patients with AIH, but also in HCV and viral hepatitis D. They recognize members of the uridine glycuronosyl transferases family 1 (26, 27, 50, 51).

### Anti-Soluble Liver Antigen/Liver–Pancreas Antigen Antibodies (Anti-SLA/LP)

Anti-SLA/LP have the highest specificity for AIH among all AIH-related autoantibodies (9). However, they are present in only about 10–20% of patients. Due to their high specificity, anti-SLA/LP should be tested routinely when AIH is suspected or in any case of unclear elevation of liver enzymes. Anti-SLA/LP cannot be detected by IFT and need to be tested for by ELISA or Western blot (52). The cytosolic autoantigen targeted by anti-SLA/LP was described independently by different groups and has been characterized later on as *O*-phosphoseryl-tRNA:selenocysteine-tRNA synthase (SepSecS), a synthase (S) converting *O*-phosphoseryl-tRNA (Sep) to selenocysteinyl-tRNA (Sec) (20, 53–58). Anti-SLA/LP belong to the IgG1 subtype of immunoglobulins and recognize an immunodominant epitope at the carboxy terminus of the SepSecS protein (59). Interestingly, the epitopes of SepSecS recognized by anti-SLA/LP-antibodies overlap with CD4+ T cell epitopes. This points to a relevant pathogenetic role of SepSecS for the subgroup of anti-SLA/LP-positive AIH patients (60). Anti-SLA/LP have been associated with the presence of a subtype of ANA (anti-Ro52) (61). Unexplained adverse pregnancy outcomes in AIH were highly associated with the presence anti-SLA/LP and anti-Ro52, potentially due to antibody-induced congenital heart block (62).

### Other Antibodies Associated With AIH

#### Anti-Liver Cytosol Type 1 Antibodies (Anti-LC1)

Anti-LC1 antibodies directed against liver cytosol 1 (anti-LC1) target epitopes of the enzyme formiminotransferase cyclodeaminase (63). They are present in about 30% of type 2 AIH patients, alone or in combination with anti-LKM-1 (64, 65). In IFT, anti-LC1 stain hepatocytes but spare the centrilobular areas of the liver. By contrast, anti-LKM-1 stain hepatocytes throughout the liver lobule. When both antibodies are coexistent, anti-LKM-1 cover the spared areas of anti-LC1. Thereby, anti-LKM-1 can mask the presence of anti-LC1. Solid-phase assays help to identify anti-LC-1 (65). When anti-LC1 are the sole antibodies being detected, they strongly support the diagnosis of type 2 AIH. However, anti-LC1 are not AIH specific and can also be detected in patients with HCV (64).

#### Antineutrophil Cytoplasmatic Antibodies With a Perinuclear Staining Pattern (p-ANCA)

The presence of p-ANCA can support the diagnosis of AIH, especially in the absence of other autoantibodies (9). However, p-ANCA can also be detected in chronic viral hepatitis, inflammatory bowel disease (IBD), PSC or microscopic polyangiitis, and eosinophilic granulomatosis with polyangiitis (66–69). p-ANCA mainly react with myeloperoxidase. Atypical p-ANCA (p-ANNA), which are characterized by the retention of a perinuclear staining on formaldehyde-fixed neutrophils seem to be more specific for autoimmune liver diseases and IBD (9).

#### Anti-Asialoglycoprotein Receptor Antibodies (Anti-ASGPR)

Anti-ASGPR can be detected in 24–82% of AIH patients, depending on the diagnostic assay used (70). Anti-ASGPR target a liver-specific membrane receptor and seem to correlate with histological activity (70). However, anti-ASGPR are not disease specific and are also present in patients with chronic viral hepatitis or PBC.

### AMA in Patients With AIH

Since IFT on the abovementioned rodent tissues allows the simultaneous detection of autoantibodies for different autoimmune liver diseases, PBC-specific AMA are occasionally detected during the diagnostic workup for AIH. In this case, a variant syndrome of AIH with features of PBC should be suspected, often indicated by persistent elevation of cholestatic liver enzymes after the AIH component has reached remission. In some rare cases, AMA develop during the course of classical AIH with a novel elevation of cholestatic liver enzymes (2). In other rare instances, AMA can be detected in AIH patients without any other laboratory or histological signs of concomitant PBC. It is unclear whether this is an epiphenomenon, whether these cases represent a subform of AIH or a very early stage of an AIH variant syndrome with additional PBC (71, 72). Additional treatment with UDCA should be decided upon on an individual basis. In acute AIH, AMA may be present as an unspecific sign of acute liver damage, and they usually disappear over time (73).

### Pathogenetic Role of Autoantibodies in AIH

It is unclear whether autoantibodies substantially contribute to the pathogenesis of AIH (Table 1). Most knowledge on a potential pathogenetic role of autoantibodies in AIH derives from analyzes of anti-LKM. The cross-reaction of anti-LKM with homologous regions of CYP 2D6 and HCV hints at a viral infection being a possible trigger for type 2 AIH. Molecular mimicry has been proposed for several autoimmune diseases. However, it has not been convincingly shown that an acute viral infection is truly the disease initiating event for a substantial number of AIH patients. For type 2 AIH patients, an abnormal expression of CYP 2D6 as the respective autoantigen of anti-LKM has been detected on the surface of hepatocytes (74). It is unclear, how autoantibodies in other types of AIH are able to target intracellular antigens. The release of nuclear or cytosolic antigens due to hepatocyte damage has been proposed as one possible mechanism. Another area of uncertainty is the fact that almost all of the antigens addressed by antibodies in AIH are not exclusively expressed in the liver, such as CYP 2D6, which is also expressed, e.g., in the central nervous system. Also, the target antigen of anti-SLA/LP is expressed in liver, pancreas, lungs, kidneys, and activated lymphocytes, but anti-SLA/LP have only been associated with AIH (55, 57). Asialoglycoprotein receptor (ASGPR) is a hepatic C-type lectin expressed on the sinusoidal surface of hepatocytes physiologically and on the canalicular membrane of hepatocyctes under inflammatory conditions (70). A T cell-mediated immune reaction, overlapping in its targeted epitopes with the humoral immune response, has been identified for ASGPR, for CYP 2D6 (75, 76) and for SepSecS, the molecular target of anti-SLA/LP (60, 77).

## Autoantibodies in PBC

### Introduction

#### Diagnosis of PBC

Primary biliary cholangitis is a chronic non-suppurative granulomatous inflammation of the small intrahepatic bile ducts (2). The first description of this disease dates back more than 100 years (78) and historically, PBC was often diagnosed only *postmortem* (79). Around 90% of all affected patients are female, and the vast majority (>90%) display autoantibodies. Clinically, PBC leads to elevation of cholestatic parameters and often a selective IgM elevation. In addition, many patients will have elevated cholesterol levels. Autoantibodies play a major role for the diagnosis of PBC: European Association for the Study of the Liver (EASL) recommends that in adult patients with chronic cholestasis and no likelihood of systemic disease, a diagnosis of PBC can be made based on elevated ALP and the presence of AMA at a titer of more than 1:40. In the absence of AMA, PBC-specific ANA can serve to diagnose the disease (2) (Table 1).

#### Autoantibodies in PBC

Autoantibodies, and especially AMA, are a hallmark of the disease: The first tests for detection of AMA were developed in the 1960s (80) and facilitated diagnosis and timely treatment later on. The development not only of refined testing for AMA but also the addition of other PBC-defining autoantibodies to the repertoire has led to an increasing proportion of PBC patients displaying autoantibodies and likely also to earlier diagnosis. Today, even non-invasive testing for AMA seems feasible as a recently published approach to test AMA in saliva has demonstrated (81). It remains unclear whether these autoantibodies are pathogenetically relevant. There has been some debate about AMA being responsible for fatigue in PBC and trials to evaluate the effect of an anti-CD20 treatment are on the way (82). In patients with suspected PBC and negative AMA-testing, PBC-defining ANA should be sought (Table 1).

In all patients with unexplained chronic cholestasis or suspected PBC, autoantibody testing should be performed using IFT on rodent tissue and confirmation of fluorescence pattern using Hep-2 cells (see above) (2) Approximately 10% of PBC patients will not display AMA (2, 83). In these patients, diagnostic accuracy is improved by testing for PBC-specific nuclear autoantibodies, which will be present in approximately half of the AMA-negative patients (84). In addition, some of the nuclear antibodies have been associated with prognosis (see below); therefore, antibody pattern may in clinical practice help to risk stratify patients.

### Antimitochondrial Antibodies

Antimitochondrial antibodies have initially been described in patients with PBC by Dame Sheila Sherlock (80). These antibodies are directed against the pyruvate-dehydrogenase E2 (PDC-E2) (85) subunit located in the inner mitochondrial membrane. In IFT, AMA lead to a typical cytoplasmic staining in rat liver and kidney slices, but they are often also visible on HEp-2 cells. The vast majority of PBC patients (exceeding 90%) (86) display these antibodies (87), and they may occasionally be found years preceding an elevation of liver enzymes (see below). Increasingly often, AMA are detected, e.g., due to rheumatological screens in patients with normal liver enzymes (88, 89), usually at lower titers than in patients with overt PBC (90). A considerable proportion of patients with positive AMA but no signs of PBC will likely develop symptomatic PBC at some time point and therefore these patients should be followed clinically and biochemically (91, 92). Current data and guidelines do not support treatment in AMA-positive persons without any elevated liver enzymes or signs of PBC (2, 83). Several subtypes of AMA have been identified (traditionally named M1–M9). In this historical classification, antibodies directed against M2, M4, M8, and M9 are associated with PBC. Nowadays, combined peptides covering these antigenic regions have been constructed (93) and are widely used in confirmatory solid-phase tests.

Antimitochondrial antibodies can be comprised of IgG, IgA (94), and IgM antibodies (95) or a combination thereof, whereby AMA of the IgG3 subtype and IgA directed against PDC-E2 have been associated with more severe disease (96). In a subgroup of patients, AMA seem to be solely comprised of IgM antibodies (REF). To date, it is unclear, whether testing systems should include the possibility to detect IgM antibodies.

AMA on IFT may be confused with other cytoplasmatic antibodies (e.g., cardiolipin antibodies), therefore, it is advisable to perform a confirmatory test using one of the ELISA or Western blot test systems, which include several of the known PDC epitopes.

Primary biliary cholangitis-specific autoantibodies should be sought in patients with chronic (>6 months) elevation of cholestatic liver enzymes. Of note, patients with acute liver damage of any kind or liver failure may also display AMA (73). These usually disappear with the resolution of the acute disease.

### Anti-sp100 Antibodies

In patients without AMA and persisting suspicion of PBC, ANA should be sought by using IFT in HEp-2 cells. These antibodies will be present in around 30–50% of AMA-negative PBC patients and interestingly, have been described to be inversely correlated to the development of fibrosis (97). These ANA have so far have not been associated with any specific disease features. They are moderately specific for PBC and can rarely be detected in rheumatological diseases and in acute hepatitis of any origin (33, 98, 99). Anti-sp100 result in a typical pattern of “nuclear dots” in IFT on HEp-2-cells (100); a confirmatory test (ELISA and Western Blot) is available (101).

### Anti-gp210 Antibodies and Anti-Lamin-B-Receptor Antibodies

A subgroup of patients with PBC may display anti-gp210 or, rarely, anti-lamin-B-receptor antibodies in addition to AMA or even exclusively. Anti-gp210 (102) and anti-lamin-B-receptor (103) result in a “nuclear rim” pattern on IFT using HEp-2 cells, where anti-lamin-B-receptor antibodies usually result in a smooth staining and the anti-gp210-antibody appears as discontinuous staining on IFT. For anti-gp210, a confirmatory test is widely available; confirmatory testing for anti-lamin-B-receptor is subject to specialized laboratories. These antibodies are present in around 20% PBC patients, and in around 30–50% of AMA-negative patients and are considered specific for PBC. In addition, an unfavorable course of PBC has been associated with the presence of anti-gp210 in several studies (99, 104, 105), and a higher incidence of hepatocellular carcinoma has been reported in this patient cohort (106). Therefore, patients with these antibodies may warrant special attention in disease surveillance (83).

### Anti-Centromere Antibodies

Anti-centromere have been described in patients with systemic sclerosis but may occasionally occur in PBC patients without features of systemic sclerosis. Clinically, anti-centromere have been associated with increased portal hypertension in these patients (99) and a poor prognosis (107, 108). Anti-centromere have recently also been associated with the development of kidney disease in PBC patients (109).

### Anti-Kelch-Like 12 Antibodies, Anti-Hexokinase1 Antibodies

Recently, two new autoantibodies, anti-Kelch-like 12 and anti-hexokinase1 have been described in PBC (110). So far, no clear associations with disease activity or clinical course have been established, and their prevalence in larger populations has yet to be established.

### Antibodies Found in Patients With PBC and Additional Signs of AIH

Features of AIH may be present in 10–20% of patients who present with PBC. There are no generally accepted criteria to define these variant syndromes. In most patients, both diseases manifest simultaneously and in these patients, the Paris criteria have been established and endorsed by EASL to indicate additional AIH in a PBC patient (2, 111). AIH can be diagnosed if two of three criteria are present: (1) elevation of ALT levels >5 times upper limit the normal (ULN), (2) elevation of serum IgG levels >2 times ULN or positive anti-SMA, and (3) moderate to severe interface hepatitis on histology. In addition, the presence of anti-SLA/LP and anti-dsDNA may raise the suspicion of AIH in patients with PBC (112–114).

## Autoantibodies in PSC

### Introduction

#### Diagnosis of PSC

Primary sclerosing cholangitis is a cholestatic liver disease of unknown origin that is characterized by progressive multifocal strictures of the extra- and/or intrahepatic bile ducts (115, 116). PSC is strongly associated with a unique phenotype of IBD, mostly presenting as a mild pancolitis (117). While the onset of PSC is often insidious, the natural course leads to liver cirrhosis, hepatobiliary malignancies, colon cancer, and episodes of superimposed bacterial cholangitis (118). This results in death or liver transplantation after a median time of approximately 15–20 years (119, 120). PSC is usually characterized by a cholestatic profile of liver enzymes with a leading elevation of alkaline phosphatase. The diagnosis is nowadays established by magnetic-resonance cholangiography, since invasive direct cholangiography using endoscopic retrograde cholangiopancreatography is associated with markedly more complications, although it may yield a slightly higher sensitivity with regard to small bile ducts and the extrahepatic duct (121). Liver biopsy is usually not required to establish the diagnosis (122), unless concurrent AIH or small-duct PSC is suspected (123).

#### Autoantibodies in PSC

Although various serum antibodies have been described in patients with PSC, immune serology in PSC is generally considered unspecific and therefore of limited value to establish the diagnosis (124, 125) (Table 1). The most prevalent serum antibodies in PSC are p-ANCA, which can be found in up to 93% of patients, followed by ANA (8–77%) and anti-SMA (0–83%) (126). However, p-ANCA are also frequently found in patients with ulcerative colitis without PSC, in patients with AIH and to a lesser extent in PBC (127, 128). Due to the lack of specificity, p-ANCA can support the diagnosis of PSC only in selected patients, mainly those lacking associated IBD.

### Antineutrophil Cytoplasmic Antibodies With Perinuclear Staining Pattern (p-ANCA)

Usually, p-ANCA are demonstrated on IFT on alcohol-fixed human granulocytes. However, an experienced technician is required as evaluation of the staining patterns is challenging (129). The “atypical” pattern frequently found in sera of patients with PSC is characterized by broad inhomogeneous rim-like staining of the nuclear periphery and multiple intranuclear fluorescent foci. By contrast, “classical” p-ANCA with fine rim-like staining of the perinuclear cytoplasm are predominantly found in patients with microscopic polyangiitis (129).

The principal target antigen of p-ANCA is a matter of debate but is likely located within the cell nucleus rather than in the cytoplasm (130, 131). The most likely candidate antigen is beta-tubulin isoform 5 (TBB-5) (132). Interestingly, p-ANCA cross-react with FtsZ, which is considered an evolutionary ancestor of TBB-5 and is widely abundant in bacteria of the human intestine (132). This highlights that abnormal immune responses to commensal bacteria might be implied in the pathogenesis of PSC.

Both cytoplasmic ANCA (c-ANCA) and p-ANCA were also found in bile fluid of patients with PSC and correlated with several adverse clinical outcomes, which might further underline their pathogenetic significance (133).

The presence of p-ANCA in serum has been associated with unfavorable clinical outcomes in patients with PSC (134). However, this notion has been challenged by other authors (135). Recently, p-ANCA have been linked to a distinct clinical phenotype of PSC with p-ANCA-positive patients being younger at disease-onset and at lower risk of cholangiocarcinoma (68). Moreover, the genotypes of p-ANCA-positive patients more often comprised the strong PSC-risk alleles HLA-B*08 and DRB1*03 (68). In small-duct PSC, a more benign variant of “classical” PSC which presents with a normal cholangiogram, the prevalence, and clinical significance of p-ANCA is unclear (136).

### Antibodies to Biliary Epithelial Cells (BEC)

Few studies found antibodies of different subtypes in sera of patients with PSC directed against BEC (137–140). Levels of IgA antibodies directed against BEC were correlated with adverse patient outcomes (140). Furthermore, it has been demonstrated that antibodies against BEC and bacterial lipopolysaccharides co-activate cytokine release by BEC and therefore induce biliary immune responses (139). This provides further evidence for the involvement of microbiota in the pathogenesis of PSC. Testing for anti-BEC antibodies has not been introduced into clinical practice.

### Antibodies Found in Patients With PSC and Additional AIH

Additional features of AIH are found in approximately 5% of adult patients with PSC (141) but are prevalent in 35–65% of children with PSC (142, 143). In children and adolescents, the primary manifestation of disease may be that of typical AIH, together with cholangiographic and histological changes of sclerosing cholangitis (also called autoimmune sclerosing cholangitis) (142). Testing patients with PSC for ANA, anti-SMA using IFT and for anti-SLA/LP using ELISA should be performed when additional AIH is suspected. Liver histology is mandatory to establish suspected AIH in patients with PSC, which will affect treatment decisions since AIH should be treated with immunosuppression (1, 125).

#### Antibodies Against Glycoprotein 2 (Anti-GP2)

Recently, IgA-class antibodies against glycoprotein 2 (anti-GP2 IgA), which were formerly linked to severe types of Crohn’s disease (144), were detected in sera of patients with PSC at a prevalence of 46.7–71.5%. The presence of anti-GP2 IgA was strongly associated with large bile-duct involvement, development of cholangiocarcinoma, and increased mortality (3). Therefore, anti-GP2 might serve as a novel biomarker of risk stratification in patients with PSC. Moreover, evidence for the involvement of GP2 in immune responses of the intestinal mucosa to gut bacteria (145, 146) provides a further pathophysiological link to the recently discovered aberrant community structure of the gut microbiota in patients with PSC (147–149).

### Other Antibodies: IgG4

IgG4-associated cholangitis (IAC) can display a cholangiographical pattern similar to PSC (150). Since patients with IAC usually benefit from treatment with corticosteroids, all patients with suspected PSC should be tested for elevated serum-IgG4 levels (124). However, 10–12% of patients with classical PSC might have increased levels of IgG4 as well (150). It is unclear to date, whether these patients represent a subgroup of PSC with a different clinical presentation or course of disease.

## Conclusion

Testing for autoantibodies is required to diagnose AIH and PBC, and it may be helpful to diagnose selected patients with PSC. IFT on tissue sections and HEp-2 cells is the screening tool of choice in patients with suspected autoimmune liver disease. Immunofluorescence pattern on HEp-2 cells must be reported to correctly interpret obtained results. ELISA for anti-SLA/LP should be included in the diagnostic workup not to miss patients only presenting with these rather AIH-specific antibodies. Some of the autoantibodies may be associated with disease course, and emerging data on novel antibodies in PSC may help to elucidate the hitherto unknown pathogenesis of disease.

## Author Contributions

MS, CW-N, TL, and CS wrote the manuscript and reviewed the final version.

## Conflict of Interest Statement

The authors declare that the research was conducted in the absence of any commercial or financial relationships that could be construed as a potential conflict of interest.

## Abbreviations

AIH, autoimmune hepatitis; AMA, antimitochondrial antibodies; ANA, antinuclear antibodies; ASGPR, asialoglycoprotein receptor; BEC, biliary epithelial cells; CD, cluster of differentiation; CYP, cytochrome P450; DILI, drug-induced liver injury; DNA, deoxyribonucleic acid; ds, double stranded; EASL, European Association for the Study of the Liver; ELISA, enzyme-linked immunosorbent assay; ERCP, endoscopic retrograde cholangiopancreatography; FTCD, formiminotransferase cyclodeaminase; FTSZ, filamenting temperature-sensitive mutant Z; GP, glycoprotein; HCC, hepatocellular carcinoma; HCV, viral hepatitis C; HLA, human leukocyte antigen; IAC, IgG4-associated cholangitis; IBD, inflammatory bowel disease; IFT, indirect immunofluorescence testing; IG, immunoglobulin; LC1, liver cytosol type 1; LKM, liver–kidney microsome; MRCP, magnetic-resonance cholangiography; p-ANCA, antineutrophil cytoplasmatic antibodies with a perinuclear staining pattern; PBC, primary biliary cholangitis; PDC-E2, pyruvate-dehydrogenase E2; PSC, primary sclerosing cholangitis; SEPSECS, *O*-phosphoseryl-tRNA:selenocysteine-tRNA synthase; SLA/LP, soluble liver antigen/liver–pancreas antigen; SLE, systemic lupus erythematodes; SMA, smooth muscle antibodies; SS, single stranded; TBB-5, beta-tubulin isoform 5; UGT, uridine glycuronosyl transferase; VGT, vascular/glomerular/tubular.
